# Biological Health Markers Associated with Oxidative Stress in Dairy Cows during Lactation Period

**DOI:** 10.3390/metabo13030405

**Published:** 2023-03-09

**Authors:** Vincenzo Tufarelli, Maria Antonietta Colonna, Caterina Losacco, Nikola Puvača

**Affiliations:** 1 Department of Precision and Regenerative Medicine and Jonian Area (DiMePRe-J), Section of Veterinary Science and Animal Production, University of Bari Aldo Moro, 70010 Valenzano, Italy; 2 Department of Soil, Plant and Food Sciences, University of Bari Aldo Moro, 70125 Bari, Italy; 3 Department of Engineering Management in Biotechnology, Faculty of Economics and Engineering Management in Novi Sad, University Business Academy in Novi Sad, Cvećarska 2, 21000 Novi Sad, Serbia

**Keywords:** cattle, milk production, enzymes, oxidative stress, metabolism, biomarkers

## Abstract

This review aims to summarize and present different biological health markers in dairy cows during the lactation period. Biochemical health markers provide an indicator of how foreign chemical substances, whether external or internal, affect the animal’s health. To understand the relationship between dairy cow health issues and oxidative stress, various biomarkers of oxidative stress must be investigated. Biochemical and hematological factors play a significant role in determining the biological health markers of animals. A variety of biochemical parameters are dependent on various factors, including the animal’s breed, its age, its development, its pregnancy status, and its production status. When assessing the health of cattle, a blood test is conducted to determine the blood chemistry. To diagnose diseases in dairy animals, the blood biochemistry is necessary to determine the cause of many physiological, metabolic, and pathological problems. Observing blood alterations during pregnancy and at peak lactation may determine what factors lift oxidative stress in cows due to disturbances in feed intake and metabolic processes.

## 1. Introduction

The term “biomarker” was used for the first time in 1980 to describe the features that are intentionally measured and used to determine the effects of therapeutic interventions or to indicate the course of everyday biological processes [1]. It is possible to detect exposure to unknown chemicals in our surroundings using biomarkers [2,3]. It can be a peripheral material itself or a dissimilarity of the original exterior material following the body that has practiced it which can often be enumerated. Biochemical and hematological factors play a significant role in determining the biological health markers of animals [4]. A wide range of biochemical parameters is affected by factors such as the breed of the animal, its age, its development, and its gestation status [5].

The biochemistry of blood is estimated significantly to evaluate cattle health status [6,7]. In dairy animals, biochemical measurements are necessary to find a wide range of pathological, physiological, and metabolic problems [8,9]. It has been shown that blood glucose, cholesterol, and protein affect the fertility and reproductive cycle of dairy cattle. Among lipids, cholesterol is one of the most important [10]. Located in the bloodstream, it is considered vital for an organism’s survival [11]. Different hormones, such as estrogen, progesterone, and aldosterone, are produced by cholesterol to develop cell membranes and maintain proper body functions. By assessing variations in the parameters such as total cholesterol, high density lipoprotein (HDL), low density lipoprotein (LDL), and triglycerides (TG), variations in lipid metabolism happening in the body of cows during pregnancy and lactation can be better understood [12,13,14].

Aspartate transaminase (AST), alanine transaminase (ALT), and γ-glutamyl transferase (GGT) are serum enzymes commonly found in the tissues of the liver and considered biological health markers in case of hepatic disorders and other health issues [15,16,17]. To diagnose and understand dairy cow health issues, serum enzyme performance and other parameters must be determined [18,19]. To understand the growth of health issues in dairy cows, researchers seek biomarkers of oxidative stress [20].

Oxidative stress, by definition, is the result of an increase in oxidant production in animal body cells as a result of free radical production [21,22,23]. By releasing free radicals in the body, the body is subjected to chemical stress because it cannot neutralize or eliminate them [24]. A growing number of veterinarians are interested in finding out how different antioxidants can improve the health of animals [25]. A few biochemical markers monitor the stress caused by oxidation, but only a few diagnostic procedures evaluate total antioxidant status (TAS) [26].

When different tissues do not function as they should, malondialdehyde (MDA) is the best indicator of reactive oxygen species and the most effective detector for free radicals of oxygen [27]. The main function of the superoxide dismutase enzyme (SOD) is to change the superoxide radical into an oxygen molecule and water [28]. In the following reaction, oxygen and water are transformed from hydrogen peroxide to water and oxygen by catalase and peroxidase. Cells use catalase (CAT) to catalyze the breakdown of water into molecules of water and less reactive oxygen gas [29]. Oxidative stress can result from disturbances in antioxidant balance [30]. This type of imbalance can lead to a variety of disorders [31,32,33].

Both HDL and LDL oxidized lipids are eliminated by paraoxonase (PON1), which acts as an antioxidant molecule, and antioxidant mechanisms are thus affected by it [34]. In high milk production situations, measuring serum PON1 activity could be a helpful diagnostic tool for evaluating dairy cattle’s health [14]. Living beings possess the lipophilic antioxidants PON1 and arylesterase (ARE), which are serum esterase enzymes. A chemical reaction is catalyzed by both esterase enzymes acting together as one enzyme [35].

As an acute phase protein, ceruloplasmin (Cp) performs as an antioxidant in living beings. Acute phase proteins are imprecise biological health markers and are arbitrated by cytokines [36]. These proteins’ levels in serum modify noticeably in an acute phase. Vitamins and trace elements as antioxidants can defend the body from free radicals directly by hunting these radicals or indirectly by slowing down the enzymes’ activity of oxidation [37].

In their one-year life cycle after adulthood, dairy cattle have to pass various physiological changes. During such physiological stages including breeding, pregnancy, fetus growth and development, parturition, and lactation, dairy animals are highly influenced by changes in hormones [38]. Among these hormones, thyroid hormones (T3 and T4) directly influence the cattle’s metabolic activities, mainly enhancing the speed of metabolism of approximately all tissues [39]. Energy metabolism having main components such as lipids and carbohydrates is modulated by thyroid hormones. Cortisol is considered the best biomarker to determine stress status [40,41].

As an investigative practice, serum biochemical analyses are a reliable way to obtain information about dairy animals’ metabolic and health status. By doing so, we can compare the standard values of healthy animals with those of animals with detrimental health statuses [42]. To gain a better understanding of the role of oxidative stress on animal welfare and production, Celi and Gabai [43] encouraged other scientists to recognize the biomarkers of protein oxidation in veterinary medicine.

Based on the previously presented, the objective of this review was to summarize and present the biological health markers of cows during lactation.

## 2. Production of Free Radicals

Oxidative stress is an energetic ground of research in the veterinary field and is related to numerous diseases. Nowadays, studies have been paying thoughts to oxidant and antioxidant status that have an effect on dairy cattle during the gestation and lactation period [44,45]. Oxidation involves the loss of electrons and reduction is the addition of electrons. The number of electrons increase or decrease when involved in an electron transfer which raises due to oxidation and reduction. Reactive oxygen species is a common term which includes both oxygen radicals and some non-radicals, which take steps as oxidizing agents and are simply converted into radicals [46,47]. Free radicals are very reactive atoms or molecules having one or more than one unpaired electron. Biologically related free radicals are groups of atoms generally having oxygen or nitrogen with an unpaired number of electrons. When a bond is broken during a chemical reaction, free radicals are formed. The free radicals can attach to tissues and damage the tissues [48]. Mostly, biological compounds such as carbohydrates, proteins, and lipids are damaged. When oxygen species and nitrogen species are immediately produced, they collectively also act as free radicals [49].

During normal physiology and metabolism in tissues, free radicals such as reactive oxygen species (ROS) are constantly produced. For the generation of ATP, oxygen is utilized in mitochondria and water is formed by the reduction of oxygen, but some quantities are not reduced completely and form an oxygen intermediate compound. In living cells, the main free radical is the superoxide radical, produced in the mitochondria by the electron transport chain [50]. Free radicals produced during oxidative metabolism form fatty acid hydroperoxides. When these peroxides react with fatty acids, they generate a chain reaction which again produces free radicals. In a stress condition, macrophages are also produced as a source of free radicals. ROS is also produced by immune cells [51]. Next to free radicals and ROS, other molecules with oxidative properties are produced during metabolism, such as reactive nitrogen species (RNS) or reactive chlorine species [52].

## 3. Antioxidants and Oxidative Stress in Cows

**Figure 1:**
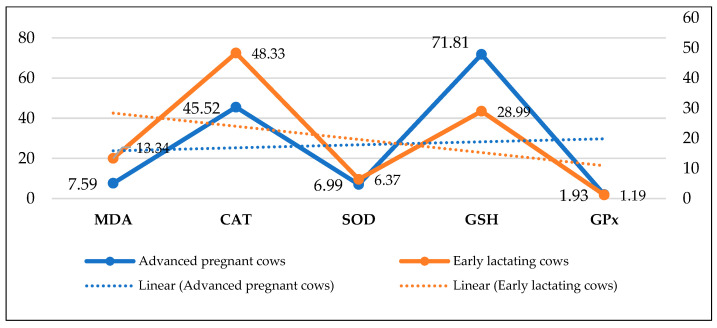
Oxidative stress and antioxidant status in advanced pregnant and early lactating dairy cows.

In oxidative damage prevention or removal, antioxidants play an important role [53,54]. By protecting the body from free radicals, antioxidants play an important role. There are two types of defense systems: enzymatic and non-enzymatic. Among the enzymes are SOD, glutathione peroxidase (GPx), and CAT, while the non-enzymatic vitamins are C, E, and selenium [55,56]. A weak antioxidant defense occurs when reactive oxygen species and free radical production increase greatly. By reducing antioxidant defenses, biological molecules and normal physiological and metabolic functions are damaged. The formation of reactive oxygen species occurs naturally in living organisms due to the release of free radicals. Normally, prooxidants and oxidants balance each other, but when the equilibrium is disturbed, harmful effects result. In cattle, oxidative stress occurs due to a decrease in antioxidant levels near the time of parturition [57,58]. Figure 1 shows the oxidative stress and antioxidant status in advanced pregnant and early lactating cows [59].

A disturbance and disorderly metabolic process may occur due to ROS damage during oxidative stress, such as damage to lipids, proteins, or DNA. Furthermore, ROS may alter cellular membranes or other components in addition to causing damage to lipids and other macromolecules [60]. This means oxidative stress does not present a precise clinical picture and does not act as a disease. As a result of oxidative stress, animals with high metabolisms are likely to develop other metabolic diseases [61]. Therefore, the role of antioxidants and oxidative stress in animals is well understood thanks to enzymatic and non-enzymatic substances.

## 4. Oxidative Stress during Lactation in Cows

**Figure 2:**
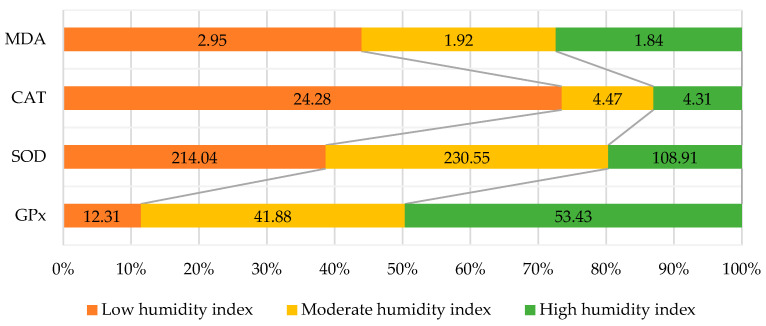
Effect of temperature–humidity index (THI) on antioxidant status of lactating cows.

Oxidative stress can weaken dairy cattle to several diseases and metabolic disorders during lactation. As a result, the physical condition and reproductive capability of dairy cows are affected [62]. During lactation, energy demands are increased, so this is the stressful stage with increased metabolic activities. The normal metabolism of the animals changes and stress is produced, thus metabolic disorder takes place. In addition, during the late period of pregnancy and the first stage of lactation, oxidative stress progresses in a negative energy balance (NEB) [47]. Dairy cows experience a drastic change in metabolism around parturition. Daily dry matter (DM) intake declines up to 30%, and at the same time before lactation, energy demand raises leading to NEB. This enhances metabolism harshly, resulting in a raised production of ROS and RNS. It is also well-known that dairy cows suffer from increased oxidative stress in late gestation and early lactation can be measured by a rise in thiobarbituric acid reactive substances (TBARS) including MDA [63]. The start of lactation is an important factor for free radicals production [64], mostly a negative energy balance developed in lactating animals that have starved conditions. A negative energy balance also develops in some diseases in which oxytocin is produced [65], while environmental conditions can also affect the antioxidant status [66] of high milk-producing dairy cattle (Figure 2).

## 5. Biological Health Markers of Cows in the Lactation Period

For monitoring the animal’s health and reproductive condition, metabolic profiles are important to review the oxidants and antioxidant substances [67].

### 5.1. Serum Biochemistry and Liver Enzymes in Dairy Cows

A high intensity of energy is consumed by the gravid uterus for the growth and development of the fetus. During late pregnancy, the glucose requirement for the gravid uterus increases and there is also a greater requirement for lactation, demanding major adjustments in the production of glucose and use in the maternal liver, adipose tissues, and skeletal muscles. The negative energy balance during lactation can raise lipolysis and diminish lipogenesis, causing the raised level of non-esterified fatty acids and β-hydroxybutyric concentration, which mobilized the fats and indicator of fatty acids mobilization [68,69,70]. An imbalance in hepatic carbohydrates occurs due to the mobilization of excessive fats and fat metabolism resulting in metabolic problems, such as ketosis and fatty liver syndrome. Economic losses can be caused by metabolic disorders in dairy farmers such as decreased milk production, treatment costs, decreased reproductive efficiency, and greater involuntary culling. Lactation stimulates stress because it is a physiological condition that adapts to metabolism in cows [14]. After calving, the body condition score loss is linked with an NEB [71]. In cows, NEB is produced by the mobilization of body reserves, because more nutrition is needed for milk synthesis. It is well known that to meet the nutritional demands of milk synthesis, dairy cows need to mobilize body reserves; awaiting nutrient intake covers the demand [71,72]. The study of Basoglu et al. [73] indicated that there was an increase in glucose VLDL, triglyceride levels before parturition, HDL levels, and cholesterol during late lactation in dairy cows. The dairy cows were tending to the fatty liver because of lower VLDL and glucose levels in early and late lactation and were inclined to hyperketonemia in early lactation because of lower insulin levels than in late lactation. Hagawane et al. [74] reported that in early and late lactating cows, blood glucose concentration was lower and significantly increased in dry cows. The downward trend of serum cholesterol was observed in dry pregnant cows as compared to lactating cows. The study of Piccione et al. [75] experimented on five healthy pregnant and lactating Holstein Friesian dairy cows. Samples of blood were collected at late gestation and early lactation during the 15, 35, and 105 days after parturition and at the end of lactation. Urea, total proteins, creatinine, albumin, total cholesterol, triglycerides, nonesterified fatty acid (NEFA), β-hydroxybutyrate, total and indirect bilirubin, calcium, phosphorus, and magnesium were determined on each sample. It was observed that the physiological phases have a significant effect on urea, creatinine, total proteins, total cholesterol, triglycerides, NEFA, β-hydroxybutyrate, calcium, and phosphorus. The study confirmed that a metabolic lactation period is more rational for the high-producing dairy cow. During the three situations such as late pregnancy, lactation, and disease, animals had undergone a negative energy status. High-yielding dairy cows during lactation undergo an NEB because energy is used for milk production and less feed intake; during the first four weeks of lactation, lipids uptake is increased by the liver thus the capacity of lipid oxidation results in a fatty liver or hepatic lipidosis. During the first stage of lactation in high yielding cows, lipid mobilization was observed, causing the liver lesion by fatty infiltration. This hepatic lesion increases the possibility of distress to the animal from other disorders such as mastitis, ketosis, hypocalcemia, and retention of the placenta more reactively or less reactively. AST and GGT appear to be the most useful for identifying hepatic disease in animals. The sensitive indicator of liver damage is the increased activity of AST; still, the level of damage is subclinical. 

Stojević et al. [76] reported the behavior of aspartate, AST, ALT, and GGT in the plasma of dairy Holstein breed cows. The lactation period was divided into three groups and the fourth one was the dry period. The first group covered the 10th to 45th day of lactation, the second from the 46th to 90th day, and the third from the 91st to the end of lactation. The dry period is considered the 4th period. In the first lactation period, AST activity was highest and in the 2nd and 3rd it was higher than in the dry period. ALT showed a significant increase from the 46th day of lactation until the dry period. ALT activity in the 2nd and 3rd periods was higher than in the dry period. In the first production period and dry period, GGT activity was statistically higher as compared to the second and third periods. On the enzymes, there was a significant influence of lactation and the dry period, and it was concluded that constant monitoring is a need for good production.

### 5.2. Lipid Peroxidation and Antioxidant Enzymes in Dairy Cows

During metabolism, reactive oxygen species are produced, and their production and balance are controlled by enzymatic and non-enzymatic defense mechanisms [77]. Enzymatic antioxidants are SOD and CAT, while ascorbate, vitamin E, and β-carotene are non-enzymatic antioxidants. Due to elevated energy demand and increased oxygen necessity during lactation, oxidative stress is produced [78]. Lactation is a physiological action; any change in biochemical positions results in complications. Bhullar et al. [79] studied lipid peroxidation, glutathione peroxidase, and superoxide dismutase behavior, finding that they were firm with the plasma level of vitamin E and β-carotene during early, mid, and late pregnancy, early lactation, and the dry period. 

On Holstein dairy cows, Sharma et al. [59] studied the stress due to oxidants and in turn antioxidant conditions during advanced gestation and early lactation. MDA was measured as a marker of lipid peroxidation and SOD, CAT, GSH, and GPx as antioxidants. During early lactation, the values of lipid peroxidation were significantly higher as compared to advanced gestation stages. In early lactation between MDA and CAT, a significant positive correlation was found. Blood glutathione (GSH) was significantly lower in early lactating cows than in the late pregnant stage. There was no significant negative correlation between lipid oxidation and all antioxidant enzymes. It is concluded that dairy cows have more oxidative stress and less antioxidant defense during early lactation than late pregnant cows. 

Konvičná et al. [57] studied the indicators of oxidative stress MDA and SOD, GPx, selenium, and vitamin E in dairy cows in late gestation and early lactation. The significantly higher MDA concentration was in one week after calving as compared to three, six, and nine weeks. During the full monitoring period, SOD activities were increased and GPx activities were decreased during one week after calving as compared to six and nine. Vitamin E was found in the lowest concentration in the first week after calving. Between MDA and SOD, GPx, and vitamin E, significant changes proved that oxidative pressure occurs during the parturition and may be a reason for the increase in the rate of metabolic disease.

### 5.3. Serum Biochemical Profile in Dairy Cows 

**Figure 3:**
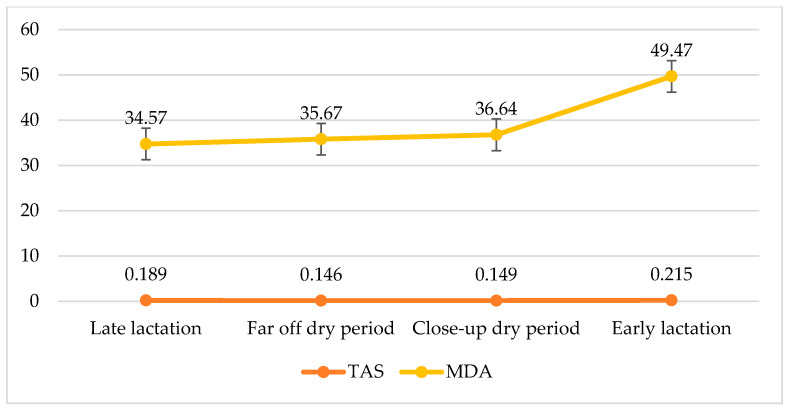
TAS (mmol/L) and MDA (µM/L) values in serum of cows in different periods.

The TAS scale has been used to determine the active balance between prooxidants and antioxidants. Oxidative stress is determined by the ratio of total peroxides to total antioxidant capacity. The serum TAS level was higher in the first week of lactation than in the cattle in pregnancy and late lactation. According to Castillo et al. [80], antioxidant activity diminishes with the passage of lactation. Castillo et al. [81] studied the values of lipid hydroperoxides and TAS in healthy cows and also studied their relationship with milk yield. The results indicated that there was a higher level of lipid hydroperoxides present in the group with a high milk yield than the other. This high oxidant compound is not accompanied by an increased level of defensive antioxidant substances. A TAS measurement gives balancing information about the metabolic status of parameters than parameters (Figure 3). Mousa and Galal [82] found that the concentration of TAS was significantly poorer before calving and the TAS concentration elevated eight weeks after calving. The decreased TAS rate before calving was synchronized with the deficiency of vitamins and minerals.

The PON1 is a calcium-dependent glycoprotein in nature that is linked with HDL [83]. PON1 acts as an enzyme that hydrolyzes organophosphorus. It has been recommended that increased oxidative stress might be associated with decreased serum PON1 activity, anti-inflammatory and antioxidative properties, and activities of PON1 give relief from physiological oxidative stress as well as contaminated environmental chemicals [84]. The liver is the site where the PON1 gene is expressed. After production, some of the PON1 residue is inside the hepatocytes and some of it is free in the blood where it is attached to HDL by association with apolipoprotein [85]. Hussein and Staufenbiel [86] studied the Cp action and copper (Cu) concentration in plasma and serum in dairy cows. In addition to this, a ceruloplasmin to Cu ratio was also observed. Serum Cu, plasma Cu, and plasma ceruloplasmin activities were increased in the fresh lactating stage. Serum ceruloplasmin showed no significant difference between fresh and early lactation. It was found that plasma Cu and plasma ceruloplasmin concentrations were increased, rather than serum Cu and Cp. Vitamin E is an antioxidant and hinders peroxidation, removes free oxygen radicals, and mixes up the break of peroxidation chain reactions by a holdback of reactive oxygen species. Near the parturition, vitamin E supplementation decreases the level of ALT, AST, and alkaline phosphatase (ALP); thus, it prevents oxidative stress by neutralizing reactive oxygen species during late pregnancy and early lactation, and the liver condition becomes better. The cows during lactation and mastitis have lowered vitamin C in milk and plasma [86]. Vitamin C scavenges the reactive oxygen species by a fast electron transfer and inhibiting lipid peroxidation, showing an important antioxidant defense next to oxidative damage. Cellular and non-cellular immunity can be increased by the antioxidant vitamins. Vitamin C has an inspiring effect on the phagocytic activity of leukocytes and the formation of antibodies. Vitamin C with the phagocytic cells uses free radicals and reactive oxygen species to destroy the pathogen. Thus, vitamin C defends the cells from oxidative damage [87].

### 5.4. Alterations in the Antioxidant Status of Health Markers in Dairy Cows

Ruminant medicine is relatively new in terms of assessing oxidative status. There have actually been a number of studies in cattle, sheep, and goats, but they have mostly focused on the effects of diseases, such as mastitis, pneumonia, sepsis, acidosis, ketosis, enteritis, joint disease, and retained placentas [88,89,90]. Nowadays, peripartum metabolic diseases are becoming increasingly studied in dairy ruminants, while dairy cattle blood biochemical parameters are well-established as a means of analyzing metabolic profiles [67]. Nevertheless, metabolic profile tests can serve as an effective method of discovering which areas of dairy management and nutrition require more attention [91].

Free radical damage detection has emerged as an important complementary tool for evaluating metabolic status in recent years [92]. To combat free radical accumulation, the body has sufficient antioxidant capacity under normal physiological conditions, while ROS are produced in the body as a result of aerobic metabolic pathways. Maintaining homeostasis requires an equilibrium between ROS production and neutralization [93]. It is important to know that when domestic animals are in the productive phase, oxygen free radicals are produced [94]. There are a number of biomarkers that can be used to monitor oxidative stress, which result from increased exposure to or production of oxidants. Through TAC estimation, antioxidative systems are monitored for their efficacy against ROS. Antioxidants other than enzymatic antioxidants are found in serum, such as GSH, α-tocopherol, and β-carotene [95]. Taking into account the cumulative effects of all the antioxidants present in plasma, TAC is a useful, reliable, and sensitive indicator. Additionally, TAC can be used to assess the nutritional status of calves during transportation and for measuring stress. The measurement of TACs and the levels of MDA, as major components of total body antioxidants in dairy cows, are useful in identifying their relationship to lactational stages and the dry period [91].

The major portion of the total antioxidants in the body are plasma total thiols, which serve as a marker of oxidative protein damage. Thiol compounds have a high antioxidant capacity since the sulfur atom can easily accommodate electron loss [96]. There have been reports indicating that total thiol levels are low in various physiological and pathological conditions, such as diabetes mellitus, cardiovascular disorders, kidney disorders, and neurological disorders that are caused by excessive free radical production [97,98,99,100]. A primiparous cow, or a cow in the early stages of lactation, is more susceptible to infections and metabolic diseases than a multiparous cow [101,102]. For this reason, it is vital to assess the metabolic and oxidative markers in cows to detect at-risk cows, especially primiparous or early lactating cows.

## 6. Conclusions

Free radical production, oxidative stress, and damage are great concerns when dealing with high-producing dairy cows. Oxidative stress biomarkers are considered to be useful tools to comprehend animal welfare since stress can reduce the body’s antioxidant resources and, as a consequence, lead the animal to metabolic disorders or diseases. In dairy cows, oxidative stress biomarkers undergo wide changes in production and concentration during the different stages from pregnancy to parturition and lactation. Hence, blood analyses carried out during these periods is helpful to detect the oxidative status of the animal and to identify possible strategies in order to limit the negative effects of free radicals and oxidant molecules upon health, production, and reproduction. Enzymes from the liver, blood, and microbial marker mined from the different states of dairy cattle gut by high-throughput sequencing (metagenome) should not be forgotten as important health markers as well. A growing number of researchers are interested in gaining further insight on the mechanisms by which different antioxidants can improve the health of animals and, consequently, milk yield and quality.
